# Cocoa Polyphenol Extract Inhibits Cellular Senescence via Modulation of SIRT1 and SIRT3 in Auditory Cells

**DOI:** 10.3390/nu15030544

**Published:** 2023-01-20

**Authors:** Luz del Mar Rivas-Chacón, Joaquín Yanes-Díaz, Beatriz de Lucas, Juan Ignacio Riestra-Ayora, Raquel Madrid-García, Ricardo Sanz-Fernández, Carolina Sánchez-Rodríguez

**Affiliations:** 1 Department Clinical Analysis, Hospital Universitario de Getafe, Getafe (Madrid), Carretera de Toledo, km 12.500, 28905 Getafe, Madrid, Spain; luzmar.rivas@salud.madrid.org; 2 Department Otolaryngology, Hospital Universitario de Getafe, Getafe (Madrid), Carretera de Toledo, km 12.500, 28905 Getafe, Madrid, Spain; joaquin.yanes@salud.madrid.org (J.Y.-D.); juanignacio.riestra@salud.madrid.org (J.I.R.-A.); rsanzf@salud.madrid.org (R.S.-F.); 3 Department of Medicine, Faculty of Biomedical and Health Sciences, Universidad Europea de Madrid, 28670 Villaviciosa de Odón, Madrid, Spain; beatriz.delucas@universidadeuroepa.es (B.d.L.); ramaga87@gmail.com (R.M.-G.)

**Keywords:** age-related hearing loss, cocoa, senescence, hydrogen peroxide (H_2_O_2_), Sirtuin 1, Sirtuin 3, Tumor suppressor protein p53, mitochondrial reactive oxygen species

## Abstract

Cocoa, rich in polyphenols, has been reported to provide many health benefits due to its antioxidant properties. In this study, we investigated the effect of Cocoa polyphenols extract (CPE) against oxidative stress-induced cellular senescence using a hydrogen peroxide (H_2_O_2_)-induced cellular senescence model in three auditory cells lines derived from the auditory organ of a transgenic mouse: House Ear Institute-Organ of Corti 1 (HEI-OC1), Organ of Corti-3 (OC-k3), and Stria Vascularis (SV-k1) cells. Our results showed that CPE attenuated senescent phenotypes, including senescence-associated β-galactosidase expression, cell proliferation, alterations of morphology, oxidative DNA damage, mitochondrial dysfunction by inhibiting mitochondrial reactive oxygen species (mtROS) generation, and related molecules expressions such as forkhead box O3 (FOXO3) and p53. In addition, we determined that CPE induces expression of sirtuin 1 (SIRT1) and sirtuin 3 (SIRT3), and it has a protective role against cellular senescence by upregulation of SIRT1 and SIRT3. These data indicate that CPE protects against senescence through SIRT1, SIRT3, FOXO3, and p53 in auditory cells. In conclusion, these results suggest that Cocoa has therapeutic potential against age-related hearing loss (ARHL).

## 1. Introduction

Cellular aging or senescence has emerged as an important factor in age-related diseases, and it is a potential target for new therapies [1]. Numerous examples of senescence at sites of aging pathology have been reported, as in cataracts [2], glaucoma [3], osteoarthritis [4], the diabetic pancreas [5], or age-related hearing loss (ARHL) [6]. ARHL is one of the most common and important health social problems [7]. It is characterized by an age-associated decrease of hearing that is due to the dysfunction and loss of hair cells in the Organ of Corti of the cochlea [8]. There is agreement that cumulative Reactive Oxygen Species (ROS) by mitochondrial dysfunction induce hearing loss.

Cellular senescence is a process that induces proliferative arrest on cells in response to different stressors, including DNA damage, oxidative stress, oncogene activation, and metabolic alterations [1]. Oxidative stress and DNA damage are associated with the modulation of mammalian Sirtuin (SIRT), nicotinamide adenosine dinucleotide (NAD)-dependent protein deacetylase, which participates in the regulation of numerous cellular processes, including senescence, metabolism, and cell cycle [9,10]. Although still not completely defined, growing evidence has shown that SIRT is a pivotal factor in retarding cellular senescence and prolonging organismal life.

The levels of SIRT1 and SIRT6 are reported to reduce in senescent cells of human endothelial cells, lung epithelial cells, macrophages, and mouse embryonic fibroblasts exposed to oxidants [11,12]. Furthermore, SIRT modulate cellular senescence through the deacetylation of different signaling molecules such as forkhead box O (FOXO, promote lifespan extension and stress resistance), Nuclear Factor-κB (NF-κB), or p53. SIRT1 suppresses stress-induced cellular senescence by deacetylates FOXO3, FOXO4, and p53 [13,14,15,16,17]. In addition, it has been shown that cardiac hypertrophy in mice improves by reducing cellular ROS levels through upregulation of the antioxidant enzymes catalase (CAT) and superoxide dismutase (SOD), due to SIRT3-mediated deacetylation of FOXO3 (mitochondrial sirtuin) [18,19]. Furthermore, a recent report showed that the downregulation of SIRT1 and activation of FOXO3a decreases ROS in spiral ganglion neurons and cochlear hair cells of mice [20]. All these results support the idea that Sirtuins have an important role in senescence. Thus, it would be a potential goal to be able to regulate Sirtuins expression to delay the ear aging process.

Cocoa beans and their derivatives (chocolate or cocoa powder) are important sources of polyphenolic antioxidants, with approximately 380 phytochemical compounds [21]. Cocoa contains three types of polyphenolic compounds, including proanthocyanidins (58%), catechins (37%), and anthocyanidins (4%), the main ones responsible for the antioxidant properties of cocoa [22]. Several in vitro studies showed that cocoa procyanidins and flavanols could inhibit UVC-mediated DNA oxidation and low-density lipoprotein oxidation, to alter immunological system activities through the abolition of the ROS production by leukocytes and reduce erythrocyte hemolysis [23,24,25,26,27]. In addition, polyphenolic compounds of cocoa suppress phorbol ester-induced ROS generation in HL-60 cells, and the activation of NF-κB, MAPKs, and cyclooxygenase-2 expression in a mice model [28]. In another study performed on the SH-SY5Y cell line (human neuroblastoma cells) treated with H_2_O_2_ FeSO4, significant beneficial effects of the cocoa phenolic extract by downregulation of ROS production and MAPK were reported [29]. Additionally, in vivo studies have shown a relationship between the intake of cocoa and a reduction of several biomarkers of oxidative stress. A diet supplemented with 10% cocoa has been informed to retard the loss of functional pancreas cells and the progression of diabetes by the removal of ROS and cellular apoptosis in Zucker diabetic rats [30]. It has also been studied that cocoa presents protective effects against low-density lipoprotein oxidation, decreases the levels of triglycerides, as well as cholesterol, and increments high-density lipoproteins in rats with hypercholesterolemia and in a hamster model of atherosclerosis [31,32].

Regarding human studies, some research was developed on subjects who were supplemented with cocoa. Osakabe et al. (2001) tested that a daily intake of cocoa for 14 days reduces low-density lipoprotein oxidation in normal patients [33]. In addition, it has been reported that the consumption of chocolate procyanidin-rich (80 g) decreases 2-thiobarbituric acid reactive substances level (40%) and increases total antioxidant capacity (31%) in plasma samples [34]. Finally, cocoa, along with moderate exercise, reduces cardiovascular risk in individuals with overweight and obese adults or with high systolic blood pressure [35,36].

Despite all investigations, more studies are necessary to best understand the potential benefits to the health of cocoa polyphenols against oxidative stress-related diseases, such as ARHL. The aim is to investigate the inhibitory effect of cocoa polyphenols extract (CPE) against hydrogen peroxide (H_2_O_2_)-induced cellular senescence with an approach directed at the modulation of different signaling molecules, such as SIRT1, SIRT3, FOXO3, and p53.

## 2. Materials and Methods

### 2.1. Auditory Cells Lines

The HEI-OC1, OC-k3, and SV-k1 cell lines were cultured in high-glucose Dulbecco’s Eagle’s medium (DMEM; Gibco BRL, Waltham, MA, USA) with 10% fetal bovine serum (FBS; Gibco BRL, Waltham, MA, USA) at 33 °C, 10% CO_2_, and without antibiotics [37].

### 2.2. Induction of Cellular Senescence

To induce senescence, auditory cells were treated with hydrogen peroxide (H_2_O_2_) (VWR Inc., West Chester, PA, USA) at 100 μM for 1 h, a method previously described by our group [38]. Untreated cells were considered as controls.

### 2.3. Cocoa Polyphenol Extraction and Treatment

For this study, cocoa powder with a high content of polyphenols (Chococru, London, UK) was used. Briefly, the cocoa sample was washed with 2N hydrochloric acid (for 1 h at room temperature and constant agitation) and, subsequently, with acetone-water under the same incubation conditions [39]. After centrifugation, the extract was desiccated by rotary evaporation R-14 (Büchi Labor-Technik AG, Flawil, Switzerland) and lyophilized in a LyoQuest lyophilizer (Telstar, Terrassa, Spain). The polyphenol content of the extract was quantified by BQC Phenolic Quantification Assay Kit (Bioquochem, Oviedo, Spain) based on Folin–Ciocalteau method [40] using Gallic acid as standard determined the total polyphenol content.

For the cocoa treatment, 5, 10, and 20 μg/mL of CPE were diluted in a serum-free culture medium and filtered through a 0.2-μm membrane. After 20 h of CPE treatment, a 100 μM of H_2_O_2_ concentration was applied to the auditory cells belonging to H_2_O_2_ group for 1 h to induce cellular senescence, and post-treatment analysis was performed at 24 h to evaluate the protective effect of the CPE against aging.

### 2.4. MTT Cell Proliferation Assay Kit

The cell proliferation and viability were measured using CyQUANT™ MTT Cell Proliferation Assay Kit (Invitrogen™, Waltham, MA, USA). Auditory cells were cultured in 96-well plates at 5 × 10^4^ cells/well and incubated for 24 h. Following that, cells were treated with CPE and/or H_2_O_2_. After a corresponding incubation period, 10 μL of MTT solution was added to wells. At 4 h, 100 µL of the SDS-HCl solution was added to wells for 4 h at 37 °C. The absorbance was measured at 570 nm using a FLUOstar Omega (BMG Labtech, Ortenberg, Germany). The control group (untreated) was considered as 100% viability.

### 2.5. Senescence-Associated β-Galactosidase Assay

To quantify senescence, the Galactosidase Detection Kit (Abcam, Cambridge, UK) was used after the end of treatments agreed to the fabricator’s instructions. The number of blue (marker of cellular senescence) and not colored cells were counted in a minimum of three random fields for each sample. Cells were observed in an Olympus BX51 microscopy (Olympus Corporation, Hamburg, Germany).

### 2.6. Morphological Analysis

The morphological changes and characteristics of auditory cells were analyzed for by a light microscope (Olympus CKX41) post-treatment (Olympus Corporation, Hamburg, Germany).

### 2.7. Determination of Population Doubling Rate

To measure the rate of population doublings, 1 *×* 10^5^ cells/well were plated in 6 well plates for 24 h, pre-treated with CPE and/or H_2_O_2_, and then incubated. Cells were counted post-treatments using a neubauer chamber. After the quantification, 1 *×* 10^5^ cells were replated for the next count, every 72 h, for up to 3 passages. The cell population-doubling rate was calculated by the formula: [log (No. of cells counted)—log (No. of cells seeded)]/log 2, as previously described [41].

### 2.8. Indirect Inmmunofluorescence

After treatments, the cells were fixed and blocked. Then, cells were incubated overnight at 4 °C with primary antibodies: SIRT1 (1/50), SIRT3 (1/50), 8-oxoguanine (8-oxoG) (1/50), and FOXO3 (1/100) (Abcam, Cambridge, UK). Following that, the secondary antibodies Alexa Fluor 546 and 488 (1/250; Molecular Probes, Eugene, OR, USA) were administrated to the cells. Subsequently, the nuclei cells were marked with DAPI (Sigma-Aldrich, San Luis, CA, USA). Cells were visualized on an Olympus BX51 microscope (Olympus Corporation, Hamburg, Germany), and the ImageJ program was used to quantify the fluorescence intensity of the micrographs.

### 2.9. Mitochondrial Superoxide Anion Detection

MitoSOX Red fluorochrome (Invitrogen, Waltham, United States) were used to determine the mitochondrial superoxide anions (O_2_^•−^). Cell lines were plated at 2 × 10^4^ cells per well. The MitoSOX assay was performed 24 h after the treatments, adding 2.5 μM of the MitoSOX-Red reagent to the cells at 33 °C for 45 min in the dark. The fluorescence was visualized with the Olympus BX51 microscope (Olympus Corporation, Hamburg, Germany).

Finally, to corroborate the mitochondrial localization of MitoSOX-Red, cells were then stained with MitoTracker Green staining kit (Abcam, Cambridge, UK). The mitochondria labeled can be observed by fluorescence microscopy. The fluorescence intensities (green, red, and merge) were quantified using the Image J software.

### 2.10. Quantitative Real-Time Reverse Transcription-Polymerase Chain Reaction

The miRNeasy Tissue/Cells Advanced Mini Kit (QIAGEN, Hilden, Germany) was used to extract the total RNA derived from the auditory cells following the manufacturer’s instructions. Total RNA was quantified spectrophotometrically in NanoDrop One (Ther-moFisher Scientific, Waltham, MA, USA). Afterwards, using the StaRT kit (AnyGenes, Paris, France) and Veriti Thermal Cycler (Applied Biosystems, Waltham, MA, USA), the total RNA (1 µg) was reverse-transcribed. To study gene expression, quantitative real-time reverse transcription-polymerase chain reaction was performed in the 7500 Fast real-time PCR detection system (Applied Biosystems, Waltham, MA, USA). Complementary DNA (cDNA) templates were added to Perfect Master Mix SYBRG (AnyGenes Paris, France). The transcript levels were normalized to β-actin and glyceraldehyde-3-phosphate dehydrogenase (GAPDH). Relative expression levels were calculated using the comparative “Ct” method. The specific primers determined in this study were SIRT1, SIRT3, FOXO3, and p53 (AnyGenes, Paris, France). The genes references sequences are the following: NM_011640.3 for p53, NM_019740.2 for FOXO3, NM_019812.2 for SIRT1, and NM_001177804.1 for SIRT3 (AnyGenes, Paris, France).

### 2.11. ELISA

After the end of treatments, cells were lysate in a buffer T-Per (Pierce, Thermo Scientific, United States) with Complete^TM^ Protease Inhibitor Cocktail (Roche, Basel, Switzerland). Cells proteins concentrations were measured using a BCA^TM^ Protein Assay Kit (Thermo Fisher Scientific, Rockford, IL, USA) and were adjusted at the same concentration.

Protein extracts were assayed by a different mouse enzyme-linked immunosorbent assay (ELISA) kits by the following proteins. SIRT1 was quantified using an Abcam ELISA kit (ab206983; Abcam, Cambridge, UK); SIRT3 levels were measured using Mouse Sirtuin 3 (SIRT3) ELISA Kit from MyBioSource (MBS2023250; MyBioSource, San Diego, CA, USA); and FOXO3 using a Mouse FOXO3/FOXO3A ELISA Kit (LS-F32067; LSBio company, Seattle, WA, USA) according to the manufacturer’s instructions. The plates were read on the FLUOstar Omega (BMG Labtech, Ortenberg, Germany) to 450 nm.

### 2.12. Determination of Phospho p53 Levels

The phospho-p53 levels were quantified in cell using the RayBio^®^ Human/Mouse/Rat Phospho-P53 (S15) ELISA Kit (RayBiotech, Norcross, GA, USA) according to the manufacturer’s instructions. Color intensity was read using a FLUOstar Omega (BMG Labtech, Ortenberg, Germany) to 450 nm.

### 2.13. Statistical Analysis

Statistical values were calculated by test paired samples T test using the SPSS 19.0 software (IBM, Armonk, NY, USA). Data are shown as mean ± standard deviation (SD). *p* < 0.05 values indicate statistical significance.

## 3. Results

### 3.1. Quantification of Polyphenols in Cocoa Extract

After extraction, a concentration of 574.65 μg/mL of total polyphenols in cocoa powder was obtained. The post-treatment polyphenol content of cell culture supernatants was also extracted (Table 1). The results obtained show significant differences in the content of polyphenols between those treated and not treated with CEP. Thus, cells treated or not with H_2_O_2_ had a polyphenol concentration of 0 μg/mL, while in those treated with CPE, increasing concentrations of polyphenols were obtained depending on the initial dose of CPE (Table 1).

### 3.2. Protective Effect of Cocoa Extract on Cell Viability in Senescent Cells

**Figure 1:**
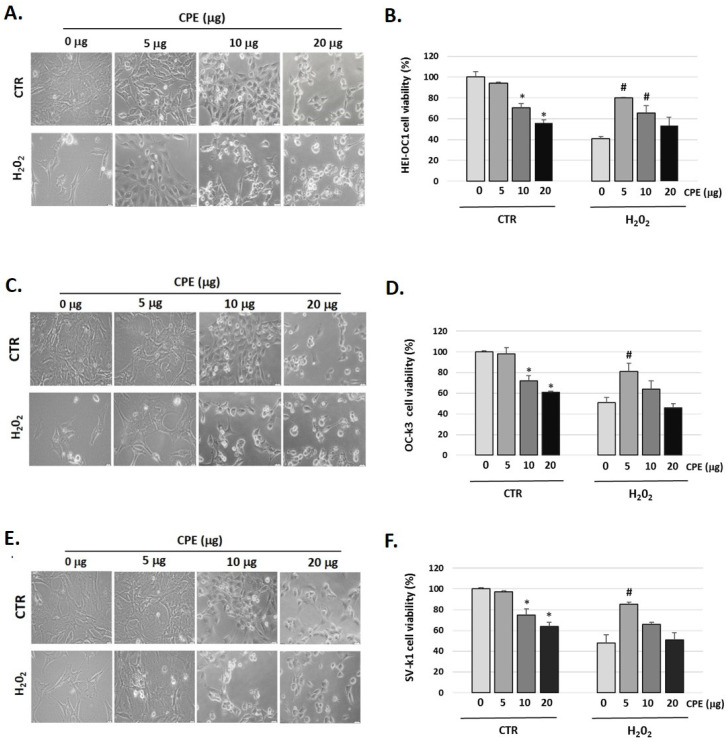
**Cocoa increases the viability of senescence auditory cells.** Representative images of morphological changes of (**A**) HEI-OC1, (**C**) OC-k3, and (**E**) SV-k1 auditory cells post-treatments. Cells were visualized under the Olympus CKX41 microscope (at 20× magnification) and the cellSens Entry imaging system. Scale bars: 100 μm. Cell proliferation and viability measured by CyQUANT™ MTT Cell Proliferation Assay Kit of auditory cells post treatments in (**B**) HEI-OC1; (**D**) OC-k3 and (**F**) SV-k1 cells. Experimental groups: CTR group (0, 5, 10, and 20 μg/mL of CPE for 20 h) and H_2_O_2_ group (CPE-pre-treatment at 0 and 5 μg/mL for 20 h and H_2_O_2_-post-treatment at 100 μM for 1 h). Results are means ± SD (n = 3). * *p* < 0.05 vs. of CTR group (0 μg of CPE); ^#^ *p* < 0.05 vs. H_2_O_2_ group (0 μg of CPE).

Figure 1 showed that H_2_O_2_ (100 μM) decreased cell viability in auditory cells to 41 ± 1.36% in HEI-OC1, to 51 ± 2.22% in OC-k3, and to 48 ± 2.10% in SV-k1 cells compared to the control group (not H_2_O_2_-treated group); this cytotoxic effect was reversed by pre-treatment with CPE (5 μg) (Figure 1B,D,F).

We also observed changes in the morphology of the three cell lines studied post-H_2_O_2_ administration (Figure 1A,C,E). In general, cells exhibited marked morphological changes characteristics of senescent cells, ranging from longer and thinner cells to ramified and enlarged cells and alterations in the organelles [42]. It should be noted that after CPE treatment to 5 μg/mL the cells recover their usual morphology (Figure 1A,C,E). CPE was used at 5 μg/mL concentrations in the following experiments, as it did not present cytotoxic effects on auditory cells.

### 3.3. Cocoa inhibits Cellular Senescence in Auditory Cells

**Figure 2:**
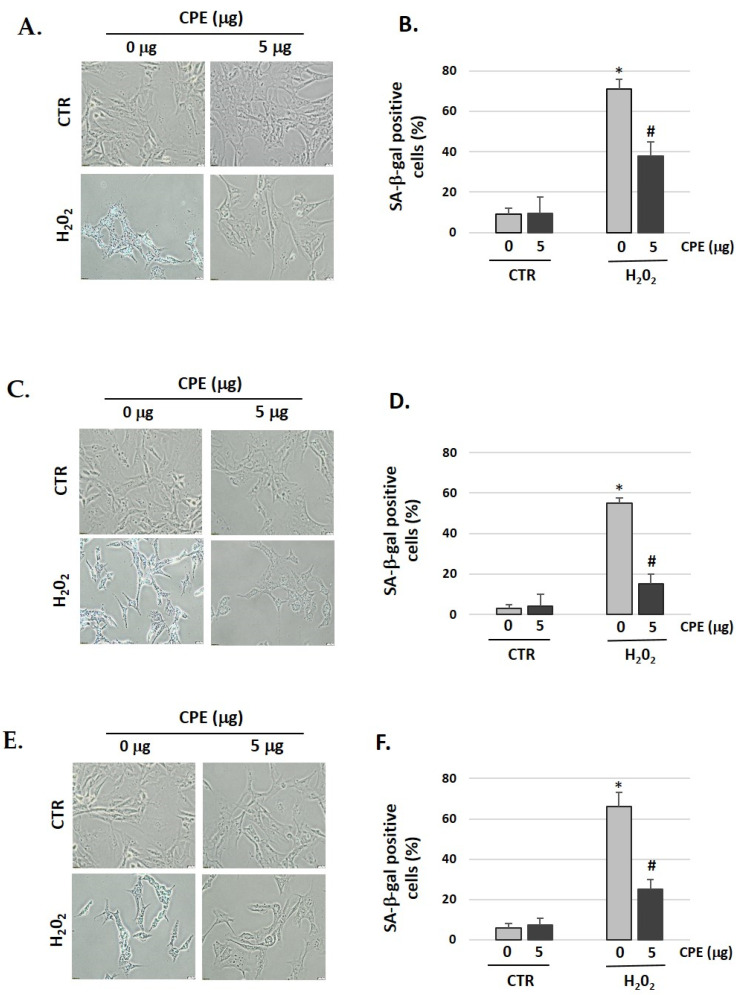
**Cocoa reduces premature senescence in auditory cells**. Representative SA-β-gal staining of: (**A**) HEI-OC1 cells; (**C**) OC-k3 and (**E**) SV-k1, post-treatment; (**B**,**D**,**F**) Percentage of SA-β-gal stained cells post-treatment. Experimental groups: CTR group (0 and 5 μg/mL of CPE for 20 h) and H_2_O_2_ group (CPE pre-treatment at 0 and 5 μg/mL for 20 h and H_2_O_2_-post-treatment at 100 μM for 1 h). Data are means ± S.D (n = 3). * *p* < 0.05 vs. of CTR group (0 μg of CPE); ^#^ *p* < 0.05 vs. H_2_O_2_ group (0 μg of CPE).

We showed that pretreatment with CPE decreased the senescent phenotype as indicated by the data of the SA-β-gal assay (Figure 2). After treatment with H_2_O_2_, 71% of HEI-OC1, 55% of OC-k3, and 66% of SV-k1 cells were SA-β-gal-positive, which was reduced to 38% in HEI-OC1, 15% in OC-k3, and 25% in SV-k1 cells in the presence of CPE (Figure 2). These data indicated that CPE protected the auditory cells against H_2_O_2_-induced cellular senescence.

### 3.4. Cocoa Extract Elevated Population Doubling Rate Post H_2_O_2_-Treatments

**Figure 3:**
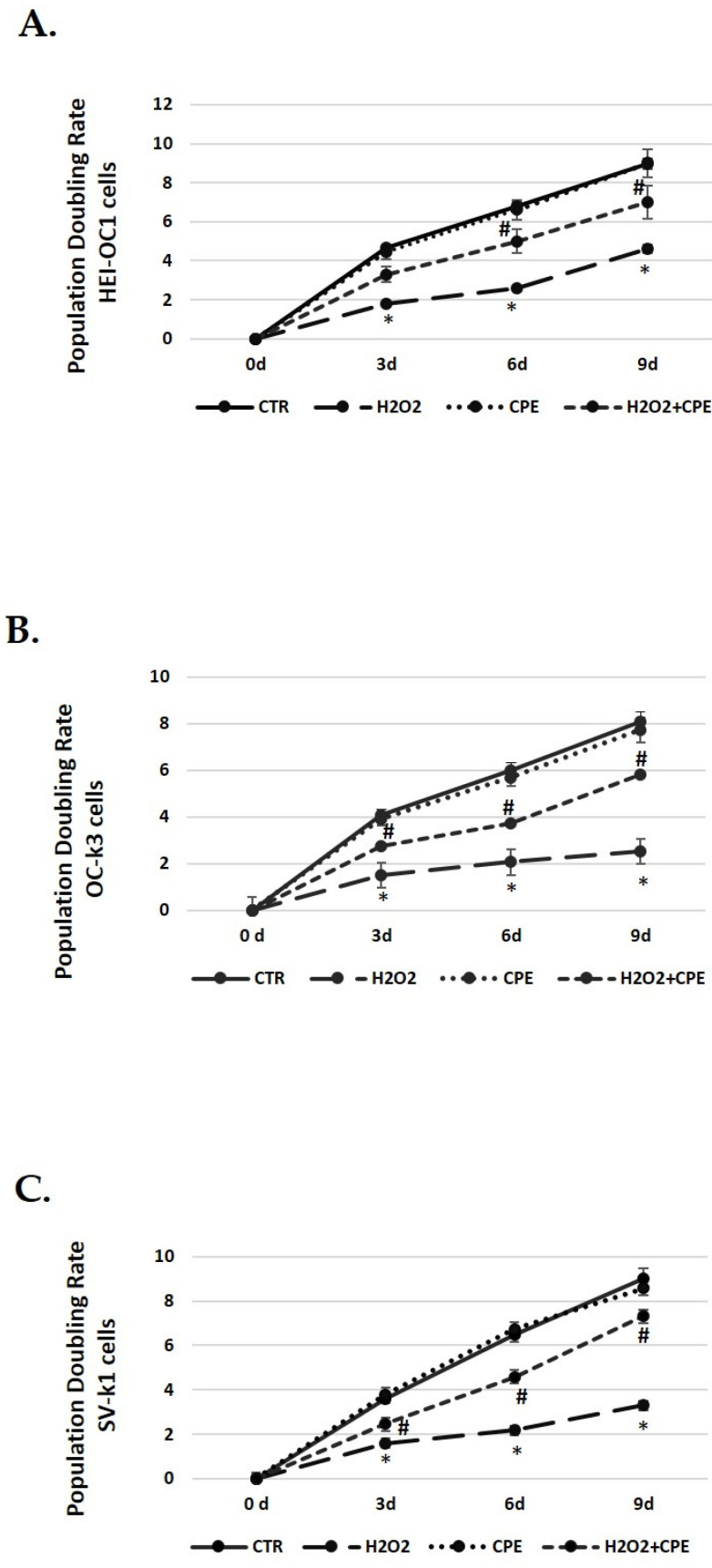
**Population doubling rate post CPE treatments increase in auditory senescent cells.** Population doubling experiments were performed post-treatments in auditory cell lines. Experimental groups: CTR group (0 and 5 μg/mL of CPE for 20 h) and H_2_O_2_ (CPE-pre-treatment at 0 and 5 μg/mL for 20 h and H_2_O_2_-post-treatment at 100 μM for 1 h). Data are means ± SD (n = 3). * *p* < 0.05 vs. of CTR group (0 μg of CPE); ^#^ *p* < 0.05 vs. H_2_O_2_ group (0 μg of CPE).

The next step was to determine the cell-doubling rate to test the cell proliferation capacity. Senescence cells exhibit lower rates, indicating a slower speed of cell growth. Controls group cells (not H_2_O_2_ treated) proliferated normally during passages, keeping the doubling rate in all cell types (Figure 3). However, a decrease in the doubling rate was obtained in cells treated with H_2_O_2_ alone (Figure 3). On the contrary, the CPE-treated cells progressively increased their duplication capacity at all passages in senescent cells (Figure 3).

### 3.5. Cocoa Increases SIRT1 and SIRT3 Expression in Auditory Senescent Cells

**Figure 4:**
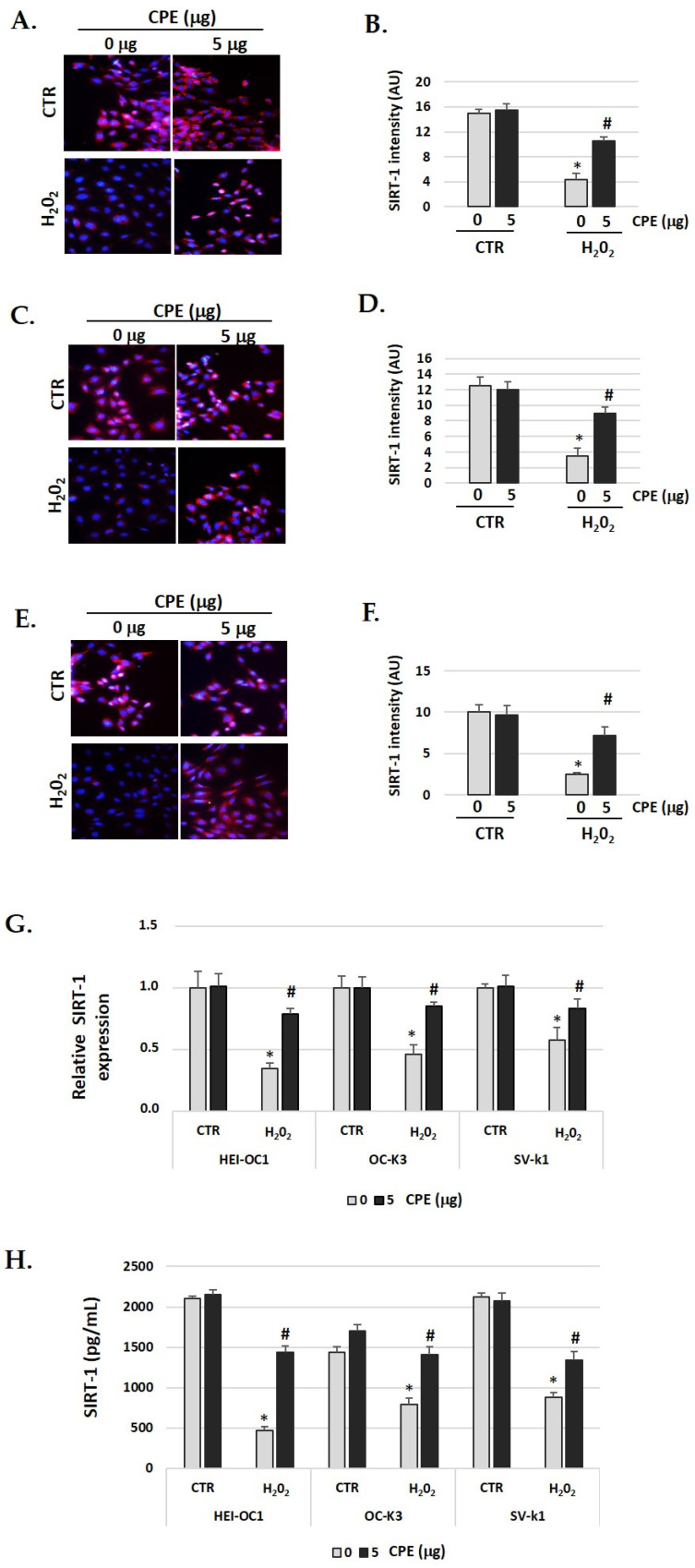
**Cocoa increase expression of SIRT-1 in senescence cells.** Illustrative fluorescence images of immunostaining of SIRT-1 in (**A**) HEI-OC1; (**C**) OC-k3, and (**E**) SV-k1 cells post-treatments. Nuclei were detected in blue fluorescence by DAPI, and SIRT-1 immunoreactivity was labeled in red fluorescence. (**B**,**D**,**F**) Graphs represent the immunofluorescence intensity analyzed by Image J of (**B**) HEI-OC1; (**D**) OC-k3 and (**F**) SV-k1 cells; (**G**) Gene expression of SIRT-1 in auditory cells cultures post-treatments; (**H**) SIRT-1 protein levels were determined by ELISA assay in auditory cells lysates post-treatments. Experimental groups: CTR group (0 and 5 μg/mL of CPE for 20 h) and H_2_O_2_ group (CPE-pre-treatment at 0 and 5 μg/mL for 20 h and H_2_O_2_-post-treatment at 100 μM for 1 h). Data are means ± SD of Arbitrary Units (AU) (**B**,**D**,**F**) and Fold change (**G**) (n = 4). * *p* < 0.05 vs. of CTR group (0 μg of CPE); ^#^ *p* < 0.05 vs. H_2_O_2_ group (0 μg of CPE). Reference genes: GAPDH andβ-actin.

The immunofluorescence identification of SIRT1 protein (red color) post-treatment shows an objective increase in CPE-treated senescent cells (Figure 4A–F) compared with H_2_O_2_-treated alone cells (*p* < 0.05). These data were congruent with the increase in the real-time PCR and ELISA assay results for SIRT1 protein (Figure 4G,H). The gene expression and protein levels of SIRT1 was significantly (*p* < 0.05) augmented in senescent cells treated with CPE.

**Figure 5:**
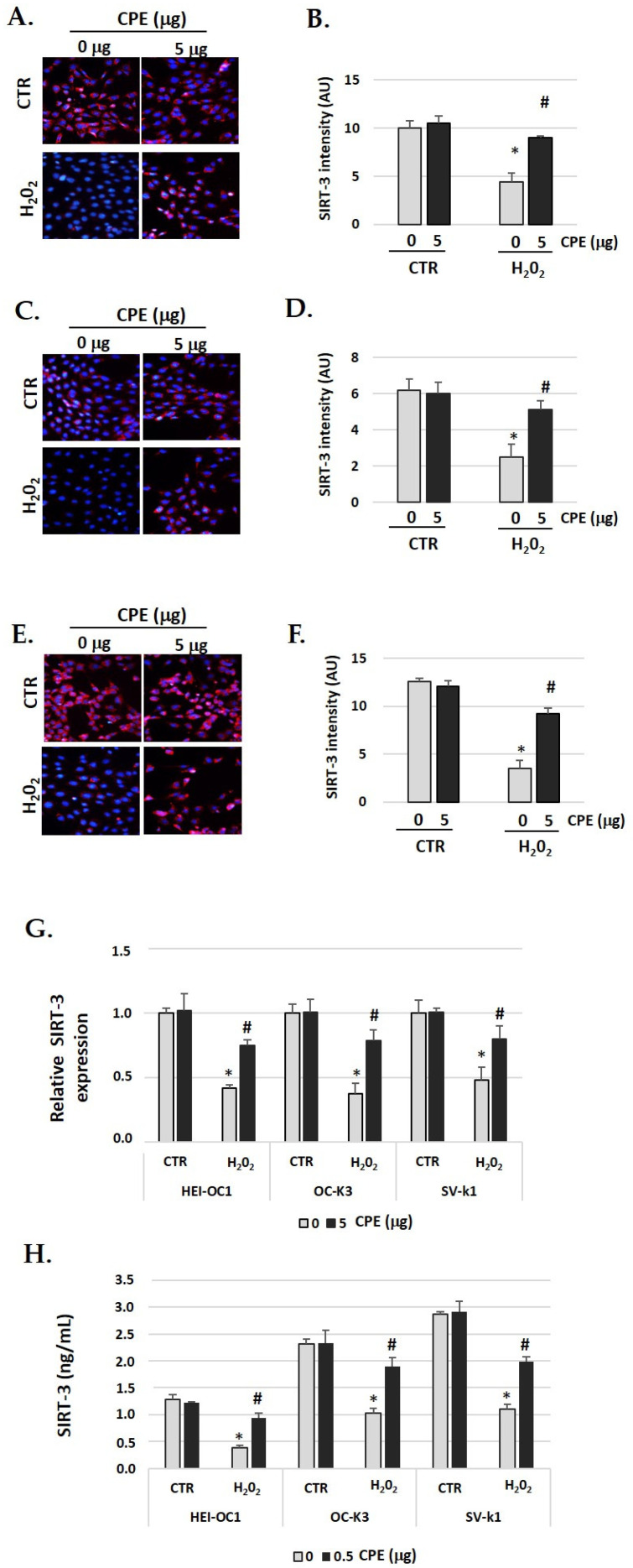
**Cocoa increase expression of SIRT-3 in senescence cells.** Illustrative fluorescence images of immunostaining of SIRT-3 in (**A**) HEI-OC1, (**C**) OC-k3, and (**E**) SV-k1 cells post-treatments. Nuclei were detected in blue fluorescence by DAPI, and SIRT-3 immunoreactivity was labeled in red fluorescence; (**B**,**D**,**F**) Graphs represent the immunofluorescence intensity analyzed by Image J of (**B**) HEI-OC1; (**D**) OC-k3 and (**F**) SV-k1 cells; (**G**) Gene expression of SIRT-3 in auditory cells cultures post-treatments. (**H**) SIRT-3 protein levels were determined by ELISA assay in auditory cells lysates post-treatments. Experimental groups: CTR group (0 and 5 μg/mL of CPE for 20 h) and H_2_O_2_ group (CPE pre-treatment at 0 and 5 μg/mL for 20 h and H_2_O_2_ post-treatment at 100 μM for 1 h). Data are means ± SD of Arbitrary Units (AU) (**B**,**D**,**F**) and fold change (**G**) (n = 4). * *p* < 0.05 vs. of CTR group (0 μg of CPE); ^#^ *p* < 0.05 vs. H_2_O_2_ group (0 μg of CPE). Reference genes: GAPDH and β-actin.

SIRT3 identification had similar behavior to SIRT1 (Figure 5A–F). In auditory cells, red fluorescence increased in the senescent group treated with CPE was compared with non-treated senescent cells. The fluorescence was quantified by Image J software, and the differences were statistically significant (*p* < 0.05) (Figure 5G). The real-time PCR and ELISA assay show similar data, with a significant increase in SIRT3 expression when the senescent cells were treated with CPE (Figure 5G,H). CPE at 5 μg/mL shows a statistically significant increase in SIRT3 expression (*p* < 0.05).

In both cases, SIRT1 and SIRT3, no significant differences were found in the CTR group between cells treated or not with CPE (Figure 4 and Figure 5).

### 3.6. Cocoa Attenuated mtROS Generation in Senescence Auditory Cells

**Figure 6:**
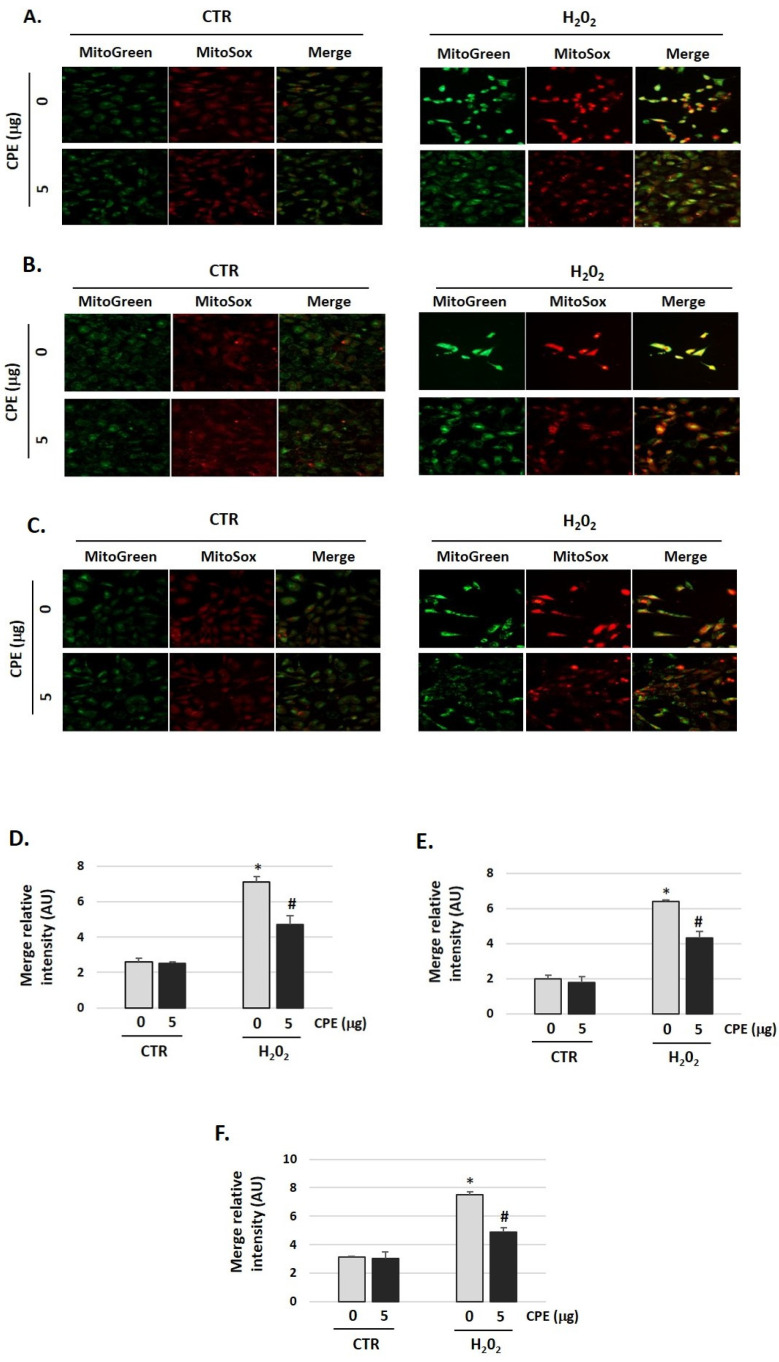
**Cocoa reduced mitochondria-derived superoxide production in senescence cells.** (**A**) Representative fluorescence images of MitoTracker Green (mitochondrial marker), MitoSOX red (mitochondrial superoxide marker), and merge (yellow) in (**A**) HEI-OC1; (**B**) OC-k3, and (**C**) SV-k1 cells. Treatments groups: CTR group (0 and 5 μg/mL of CPE) and H_2_O_2_ group (CPE-pre-treatment at 0 and 5 μg/mL for 20 h and H_2_O_2_-post-treatment at 100 μM for 1 h); (**D**–**F**) The merge fluorescence intensity per cell was calculated using ImageJ and is shown on the graphs (**D**) HEI-OC1; (**E**) OC-k3, and (**F**) SV-k1 cells. Data are means ± SD of Arbitrary Units (AU). * *p* < 0.05 vs. of CTR group (0 μg of CPE); ^#^ *p* < 0.05 vs. H_2_O_2_ group (0 μg of CPE). (n = 3).

To evaluate the effects of CPE on mitochondrial redox status, a fluorescent probe was used for the detection of the O_2_^•−^ generated in mitochondria, the MitoSOX Red. As shown in Figure 6, H_2_O_2_ increased O_2_^•−^ production (H_2_O_2_ group) compared with the control group (CTR group), which was markedly reduced by CPE pretreatment of the three auditory cells lines (*p* < 0.05). Additionally, cells were stained with Mitotracker-Green to check active mitochondria and the total quantity of mitochondria. We found growth of the number of mitochondria in cells treated with H_2_O_2_ vs. non-treated cells. H_2_O_2_ treatment increased the mass of mitochondria (Figure 6), but the preventive treatment with CPE decreases it.

### 3.7. Cocoa Extract Induces FOXO3 Activation in Senescence Auditory Cells

**Figure 7:**
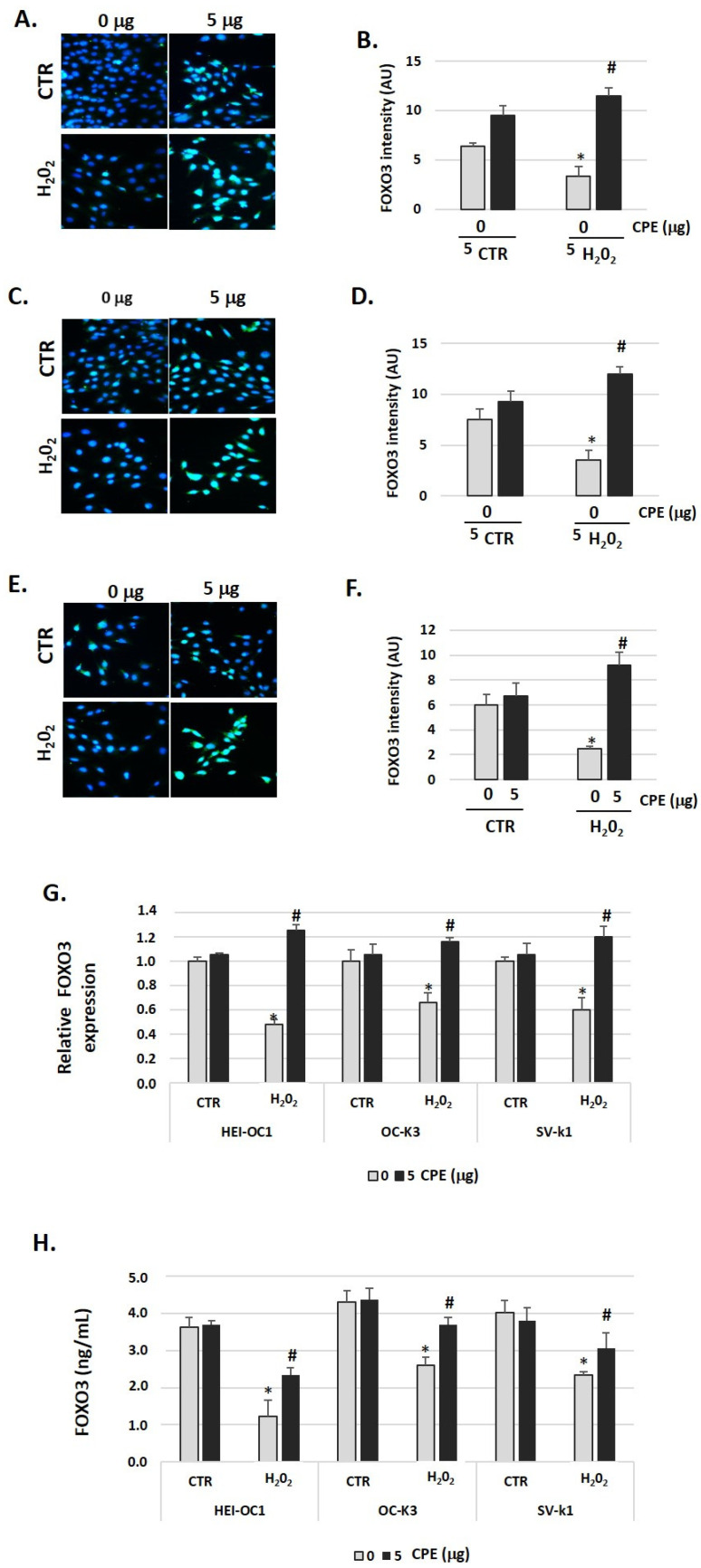
**Cocoa increase expression of FOXO3 in senescence cells**. Characteristic fluorescence images of staining for FOXO3 in (**A**) HEI-OC1, (**C**) OC-k3, and (**E**) SV-k1 cells post-treatments. Nuclei were detected in blue fluorescence by DAPI, and FOXO3 immunoreactivity was labeled in green fluorescence; (**B**,**D**,**F**) Graphs represents the immunofluorescence intensity analyzed by Image J for (**B**) HEI-OC1, (**D**) OC-k3, and (**F**) SV-k1 cells; (**G**) Gene expression of FOXO3 in auditory cells cultures post-treatments; (**H**) FOXO3 protein levels were determined by ELISA assay in auditory cells lysates post-treatments. Treatments groups: CTR group (0 and 5 μg/mL of CPE for 20 h) and H_2_O_2_ group (CPE-pre-treatment at 0 and 5 μg/mL for 20 h and H_2_O_2_-post-treatment at 100 μM for 1 h). Data are means ± SD of Arbitrary Units (AU) (**B**,**D**,**F**) and fold change (**G**) in triplicate. * *p* < 0.05 vs. of CTR group (0 μg of CPE); ^#^ *p* < 0.05 vs. H_2_O_2_ group (0 μg of CPE). (n = 4). Reference genes: GAPDH and β-actin.

To evaluate the effects of CPE treatment on FOXO3 protein expression in senescent cells, we measured its expression by immunofluorescence, real-time PCR, and ELISA assay twenty-four hours after stimulation with H_2_O_2_ or H_2_O_2_ + CPE. As shown in Figure 7A–F, H_2_O_2_ alone did not increase FOXO3 expression. In contrast, the addition of CPE together with H_2_O_2_ increased FOXO3 expression significantly (Figure 7A–F). In concordance with this data, we found a rise of FOXO3 gene expression and protein levels 24 h after treatment with CPE (Figure 7G,H).

### 3.8. Cocoa Extract Decrease p53 Expression in Senescent Cells

**Figure 8:**
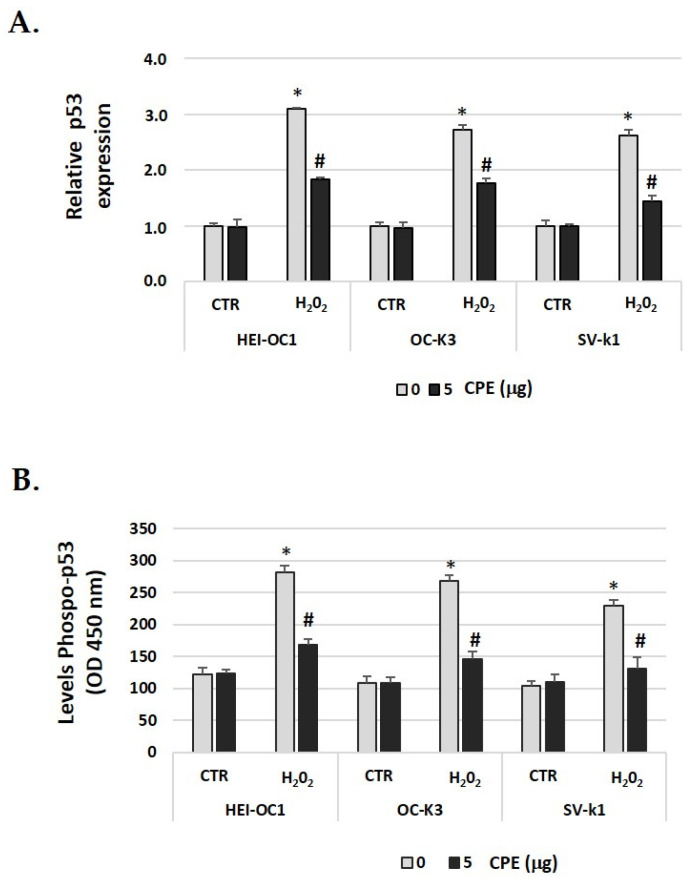
**Cocoa reduced the phosphorylation of p53 and gene expression in senescence auditory cells**. (**A**) Gene expression of *p*-53 in auditory cells cultures post-treatments; (**B**) Phospho-p53 levels were determined by ELISA assay in auditory cells lysates post-treatments. Groups: CTR group (0 and 5 μg/mL of CPE for 20 h) and H_2_O_2_ group (CPE-pre-treatment at 0 and 5 μg/mL for 20 h and H_2_O_2_-post-treatment at 100 μM for 1 h). The diagrams include the mean ± SD Fold change (**A**) and OD (450) (**B**) (n = 4). * *p* < 0.05 vs. of CTR group (0 μg of CPE); ^#^ *p* < 0.05 vs. H_2_O_2_ group (0 μg of CPE) (n = 4). Reference genes: GAPDH and β-actin.

As p53 is a key factor in the induction of senescence [43], we investigated the relationship of p53 to H_2_O_2_-induced cellular senescence in auditory cells and the effect of CPE. In Figure 8A, we showed that after H_2_O_2_ treatment, there was increased phosphorylation of p53, which was decreased after CPE treatment. We also found that p53 gene expression was down-regulated with CPE treatment in H_2_O_2_-treated auditory cells (Figure 8B). These data suggested that CPE protects cells from cellular senescence via modulation of p53 phosphorylation and expression.

### 3.9. Cocoa Prevents H_2_O_2_-Induced Oxidative DNA Damage in Auditory Cells

**Figure 9:**
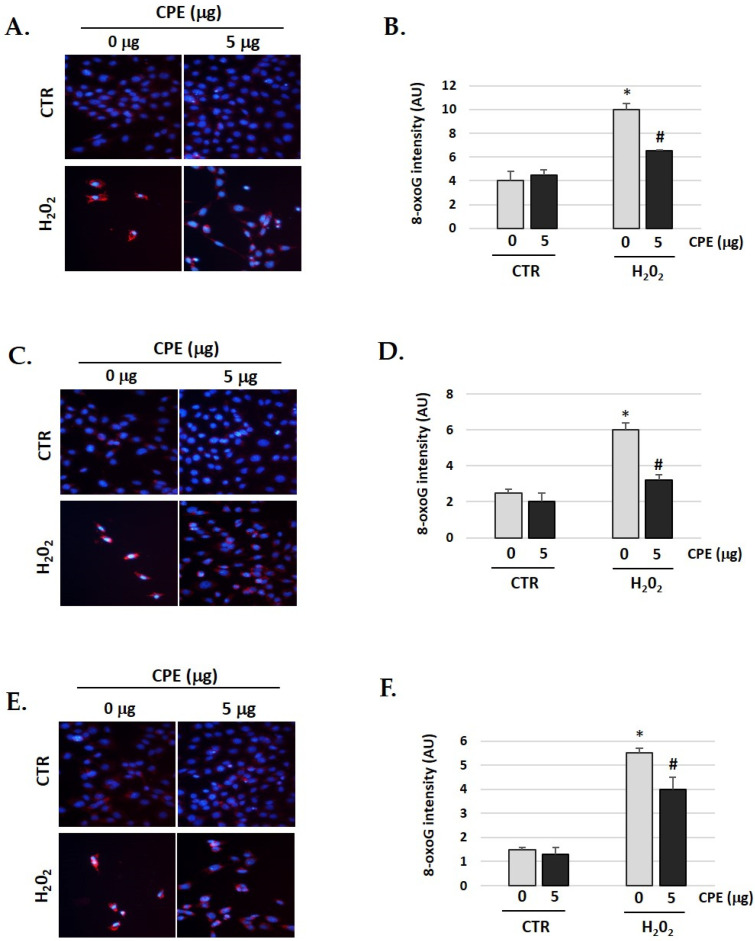
**Cocoa extract prevents H_2_O_2_-induced DNA oxidative damage in senescence cells**. Illustrative fluorescence images of staining of 8-oxoG in (**A**) HEI-OC1; (**C**) OC-k3 and (**E**) SV-k1 cells post-treatments. Nuclei were marked in blue fluorescence by DAPI and 8-oxoG was labeled in red fluorescence. Graphs represent the immunofluorescence intensity analyzed by Image J in (**B**) HEI-OC1; (**D**) OC-k3 and (**F**) SV-k1 cells. Treatments groups: CTR group (0 and 5 μg/mL of CPE for 20 h) and H_2_O_2_ group (CPE-pre-treatment at 0 and 5 μg/mL for 20 h and H_2_O_2_-post-treatment at 100 μM for 1 h). Data represent the mean ± SD Arbitrary units (AU) (**B**,**D**,**F**) (n = 4). * *p* < 0.05 vs. of CTR group (0 μg of CPE); ^#^ *p* < 0.05 vs. H_2_O_2_ group (0 μg of CPE).

A significant increase in oxidative DNA damage occurs during aging and age-related diseases. We observed the same in our senescent cells [44,45]. Therefore, we studied whether CPE has any effect on the oxidative DNA damage gendered by H_2_O_2_. As shown in Figure 9, the cells were pre-treated with CPE for 20 h before exposure to 100 μM of H_2_O_2_ for 1 h, showing a significant decrease of 8-oxoguanine (a marker of oxidative DNA damage) compared to cells treated only with H_2_O_2_. These data clearly indicate that CPE markedly protects the cells against DNA damage under H_2_O_2_.

## 4. Discussion

The polyphenols of cocoa contribute to several health benefits, such as reducing the risk of myocardial infarction, stroke, or diabetes [46,47], skin health, including acne [48], and other less explored beneficial properties of cocoa, including antimicrobial and anticancer effects [49,50,51]. However, to date, the role of cocoa in aging and age-related diseases is still unknown. The antioxidant properties of cocoa are mainly attributed to its ability to scavenge ROS, induce antioxidant enzymes, and inhibit pro-oxidant enzymes [52]. This study showed that CPE inhibits cellular senescence (H_2_O_2_-induced) and increases SIRT1, SIRT3, and FOXO3 expression, which played a pivotal role in suppressing the senescent phenotypes as well as inhibiting p53 in auditory cells.

As anticipated, CPE prevented cellular senescence, as shown by attenuation of the SA-β-gal stain and restoration of cell proliferation and morphology. A previous study by Ramirez-Sanchez et al. [53] proposed that (−)-epicatechin (cocoa product) might inhibit senescence in bovine coronary artery endothelial cells.

Our experimental results showed that SIRT1 and SIRT3, which mediate the otoprotective effects of CPE against H_2_O_2_ damage, are in agreement with the importance of Sirtuins in aging [54,55]. In particular, SIRT1 is a crucial factor in protection against cellular oxidative stress and DNA damage [56]. Our data showed that CPE increased SIRT1 expression and reduced ROS production and oxidative DNA damage in auditory senescent cells, which avoided H_2_O_2_-induced senescence. Previous research has determined that increased SIRT1 expression inhibited oxidative stress-induced senescence in human endothelial cells [57]. Zhu et al. [58] observed that Luteolin, a sort of flavonoid, in HEI-OC1 cells induces SIRT1 expression, which prevented H_2_O_2_-induced senescence; also, SIRT1 siRNA abolishes the protective effect of Luteolin on H_2_O_2_-induced DNA damage [59,60]. Polyphenols also protect cells against oxidative stress by SIRT1 overexpression [61,62,63,64]. Interestingly, different studies have highlighted that a diet rich in polyphenols has a protective function against cardiovascular, neurodegenerative, metabolic, inflammatory diseases, and cancer by increasing SIRT1 expression [65], and it has been reported that polyphenols, such as quercetin, catechins, and resveratrol, activate SIRT1 in vivo and in vitro studies [65,66,67,68]. Therefore, it would be promising to control SIRT1 activity by cocoa flavanol as a therapeutic application against oxidative stress-induced cellular senescence.

Recent studies suggested that SIRT3 is crucial to regulate mitochondrial ROS (mROS) homeostasis [69]. SIRT3 acts through the deacetylation of a variety of substrates in the mitochondrial matrix where oxidative metabolism takes place, such as the oxidative phosphorylation cycle (OXPHOS) and tricarboxylic acid (TCA). [70,71]. Moreover, it has been reported that SIRT3 functions stimulate deacetylation of enzymes implicated in mtROS homeostasis and activation of FoxO3A-mediated mitochondrial DNA (mtDNA) transcription to reduce oxidative damage [69,72,73]. Consistent with previous studies, our data indicated, for the first time in senescent auditory cells, that CPE through regulation of mtROS homeostasis, mediated by activation of the SIRT3 signaling pathway, reduces oxidative damage. The current study applied MitoSOX Red probe to detect mtROS, and thus differentiate them from ROS generated in the cytoplasm or from sources exogenous to the cell. We determined that CPE treatment decreased excessive mtROS in mitochondria of the auditory senescent cells. In agreement with our results, the study by Zhou et al. [73] showed that resveratrol attenuated tert-butyl hydroperoxide (t-BHP)-induced oxidative injury in HUVEC mediated by activation of the SIRT3 signaling pathway, which reduced mtROS production. Additionally, Zeng et al. [74], using a D-galactose-induced aging rat model that provides a mechanistic of central auditory dysfunction in ARHL, showed that decreased SIRT3 might have less ability to increase superoxide dismutase 2 (SOD2) activity, translating into an elevation in ROS generation. As a result, ROS accumulates associated with aging, inducing mitochondria dysfunction and increasing apoptosis of auditory cells, ultimately resulting in ARHL.

Considering p53 protein is a critical factor that regulates numerous pathways involved in senescence [75], we investigated whether CPE alters the phosphorylation of p53 in the auditory senescent cells. Our study showed that the phosphorylation of p53 was higher in auditory senescent cells compared with non-senescent, which was notably decreased with preventive administration of CPE. These results are in line with previous studies showing that regulation of p53 phosphorylation by Luteolin is at least partially mediated by SIRT1 in HEI-OC1 senescent cells [58] or the protective effect of Salidroside by regulation of redox and p53/p21 expression in senescence [76]. The SIRT1/p53 signaling pathway is closely related to transcriptional alterations of cell cycle inhibitors downstream of various cellular processes, such as cell proliferation and senescence [77].

## 5. Conclusions

In conclusion, the results of this study provide relevant insights supporting that cocoa polyphenol extract exerts protective effects against H_2_O_2_-induced cellular senescence. The enhancement of SIRT1, SIRT3, and FOXO3 expression by CPE plays a key role in inhibiting phosphorylation of p53 and suppressing the senescent in the auditory cells. These data present cocoa as a potential natural compound to attenuate the health problems derived from age-related diseases, such as ARHL.

## Figures and Tables

**Table 1:** Total polyphenol content in cultured cells.

	*0 μg/mL*	*5 μg/mL*	*10 μg/mL*	*20 μg/mL*
*CTR*	0	5611	9344	20,111
*H_2_O_2_ (100 μM)*	0	4834	8989	18,536

## Data Availability

Data is contained within the article.
